# Short-term clinical results of bicruciate-retaining total knee arthroplasty using personalized alignment

**DOI:** 10.1186/s12891-023-07083-5

**Published:** 2023-12-12

**Authors:** Hiroshi Inui, Ryota Yamagami, Kenichi Kono, Kohei Kawaguchi, Tomofumi Kage, Ryo Murakami, Haruhiko Nakamura, Kazuo Saita, Shuji Taketomi, Sakae Tanaka

**Affiliations:** 1 Saitama Medical Center, Saitama Medical University, 1981 Kamoda, Kawagoe City, Saitama 350-8500 Japan; 2 Department of Orthopaedic Surgery, Faculty of Medicine, The University of Tokyo, 7-3-1 Hongo, Bunkyo-ku, Tokyo, 113-0033 Japan

**Keywords:** Bi-cruciate retaining total knee arthroplasty, Personalized alignment, Unicompartmental knee arthroplasty, Patient-reported outcomes, Short-term clinical outcomes

## Abstract

**Background:**

Bicruciate-retaining (BCR) prosthesis has been introduced to recreate normal knee movement by preserving both the anterior and posterior cruciate ligaments. However, the use of BCR total knee arthroplasty (TKA) is still debatable because of several disappointing reports. We have been performing BCR TKAs with personalized alignment (PA). This study aimed to reveal the limb alignment and soft tissue balance of FA-BCR TKAs and compare the clinical outcomes of FA-BCR TKAs with those of unicompartmental knee arthroplasty (UKA).

**Methods:**

Fifty BCR TKAs and 58 UKAs were included in this study. The joint component gaps of BCR TKA were evaluated intraoperatively and the postoperative hip–knee–ankle (HKA) angle, medial proximal tibial angle (MPTA), and lateral distal femoral angle (LDFA) were measured using full-length standing radiography. The short-term clinical outcomes of BCR TKAs were compared with those of UKA using the scoring system of 2011 Knee Society Scoring (KSS) and the knee injury and osteoarthritis outcome score (KOOS) at an average of 2 years postoperatively (1-4yeras).

**Results:**

The coronal alignment values of PA-BCR TKA were as follows: HKA angle, 177.9° ± 2.3°; MPTA, 85.4° ± 1.9°; and LDFA, 87.5° ± 1.9°. The joint component gaps at flexion angles of 10°, 30°, 60°, and 90° were 11.1 ± 1.2, 10.9 ± 1.4, 10.7 ± 1.3, and 11.2 ± 1.4 mm for the medial compartment and 12.9 ± 1.5, 12.6 ± 1.8, 12.5 ± 1.8 and 12.5 ± 1.7 mm for the lateral compartment, respectively. The patient expectation score and maximum extension angle of PA-BCR TKA were significantly better than those of UKAs.

**Conclusions:**

The short-term clinical outcomes of PA-BCR TKA were comparable or a slightly superior to those of UKAs.

## Introduction

Total knee arthroplasty (TKA) is the gold standard treatment of late-stage osteoarthritis (OA); however, approximately 20% of patients are not satisfied with their surgically restored knees [1, 2].

Recently, attempts have been made to enhance knee prosthesis design to improve clinical and functional outcomes and patient satisfaction. Bicruciate-retaining (BCR) prostheses have been introduced to recreate normal knee movements by preserving both the anterior cruciate ligament (ACL) and posterior cruciate ligament (PCL). Some studies have shown that BCR TKAs may be preferable over traditional ACL-sacrificing TKAs [3, 4]. However, the use of BCR TKAs is still debatable among orthopedic surgeons because most of the reports on BCR TKAs showed clinical outcomes similar to other ACL-sacrificing TKAs and not a few studies have reported a high complication rate, including stiffness and early revision [5–9].

A complex surgical technique is one of the reasons for these poor outcomes of BCR TKA [6]. Watanabe et al. reported that the BCR using mechanical alignment showed abnormal biomechanics because of the kinematic conflict between the retained ligaments and the replaced joint surface and recommended kinematical alignment (KA) for BCR TLA to achieve sufficient ligament laxity throughout knee flexion [10]. Therefore, our surgical team has been performing BCR TKA with personalized alignment (PA), which is a modification of KA [11, 12]. If the ACL and PCL are functioning normally, the clinical outcomes following PA-BCR TKA might be comparable with those of unicompartmental knee arthroplasty (UKA), which also preserves the ACL and PCL [13].

Thus, this study aimed to reveal the limb alignment and soft tissue balance of PA-BCR TKAs and compare the clinical outcomes of PA-BCR TKAs with those of UKAs.

## Methods

This study was approved by the review board of the institution. All patients provided written informed consent.

This was a retrospective, case–control study. Between January 2019 and March 2021, 61 PA-BCR TKAs (Journey II XR; Smith and Nephew, Memphis, TN, USA) and 66 UKAs (Oxford Uni; Zimmer Biomet, Warsaw, IN, USA) were performed using an image-free navigation system (Precision N; Stryker Orthopedics, Mahwah, NJ, USA). The surgical indications for BCR TKAs were knee OA or osteonecrosis (ON) of more than two compartments, intact cruciate and collateral ligaments, preoperative flexion contracture < 15°, and preoperative deformity < 15°, and the surgical indications for UKAs were knee OA or ON of a single compartment, intact cruciate and collateral ligaments, preoperative flexion contracture < 15°, and preoperative deformity < 15°. In this study, 50 BCR TKAs and 58 UKAs met the following inclusion criteria: (1) varus deformity (2) complete data entry, and (3) minimum follow-up period of 1 year.

Preoperative patient demographics, including age, sex, weight, height, body mass index, hip–knee–ankle (HKA) angle, and range of motion (ROM), were recorded. Preoperative clinical scores were obtained using the 2011 Knee Society Scoring (KSS) system [14] and the validated version of the knee injury and osteoarthritis outcome score (KOOS) [15, 16]. The preoperative KOOS symptom score of the UKA group was lower than that of the BCR TKA group (Table 1).

**Table 1:** Preoperative demographic data

	BCR TKA	UKA	*p*-value
Number of patients	50	58	
Gender (female/male)	38/12	39/ 19	0.429
Age (years)	72.1 ± 7.4	71.7 ± 10.8	0.805
Body mass index (kg/m^2^)	25.5 ± 3.5	26.0 ± 4.4	0.533
Hip-knee-ankle angle (°)	173.3 ± 2.9	173.0 ± 3.5	0.724
Maximum extension (°)	− 3.2 ± 3.1	− 3.5 ± 3.7	0.706
Maximum flexion (°)	129.9 ± 7.9	128.5 ± 9.5	0.368
2011KSS			
Symptom	8.4 ± 6.4	9.1 ± 5.2	0.511
Satisfaction	13.0 ± 7.1	13.4 ± 7.0	0.781
Expectation	14.0 ± 1.5	13.5 ± 1.8	0.108
Activity	46.6 ± 18.7	44.3 ± 17.0	0.517
KOOS			
Pain	48.2 ± 18.4	54.5 ± 17.3	0.071
Symptom	57.9 ± 17.1	48.7 ± 19.5	0.009
ADL	58.4 ± 18.1	59.3 ± 16.4	0.802
Sports	25.4 ± 22.5	27.5 ± 21.35	0.621
QOL	27.9 ± 16.3	30.4 ± 17.7	0.452

*BCR* bi-cruciate retaining, *TKA* total knee arthroplasty, *UKA* unicompartmental knee arthroplasty, *KSS* knee society score, *KOOS* knee injury and osteoarthritis outcome score, *ADL* activities of daily living, *QOL* quality of life

All procedures were performed by five knee surgeons who used the same surgical technique. A senior surgeon (H.I.) participated in all procedures either as the chief surgeon or first assistant.

### Surgical procedure

In all patients, a paramedian approach was used, and the patella was not everted. The surgeon performed aggressive removal of osteophytes and minimal release of medial soft tissues for bone resection. The femur was made to be equal in thickness to the condyles of the femoral component. The femoral component design of the Journey TKA system was asymmetric, and the medial and lateral distal condyles were 9.5 and 7 mm thick, respectively. Therefore, using the navigation system, distal (thickness, 7–8 mm) resection was performed on the medial side, considering the cartilage wear (1 − 2 mm) based on the intraoperative findings, and distal resection of the lateral side (thickness, 7 mm) was also performed [11]. Femoral alignment in the sagittal plane aimed at 4° of flexion to avoid femoral cortex notching [17].

**Fig. 1:**
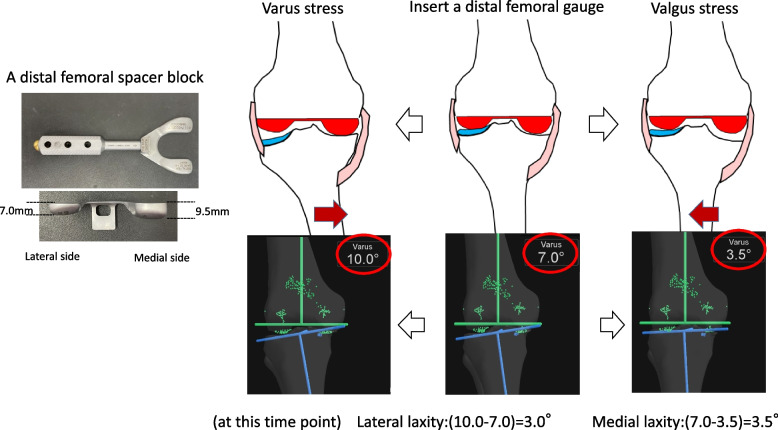
Vaus-valgus stress was applied to evaluate the medial and lateral joint laxity using the navigation system

**Fig. 2:**
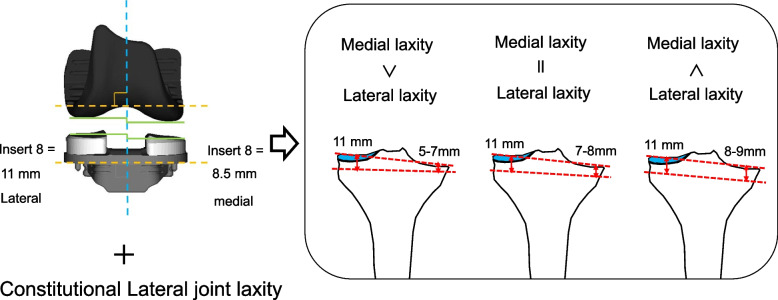
The amount of the tibial bone resection for the varus knee

Proximal tibial osteotomy was then performed. A distal femoral spacer block mimicking to the distal end of the femur component (medial 9.5 mm, lateral 7 mm) was placed on the resected distal femur, and the knee was brought into extension. Varus–valgus stress was applied to evaluate the medial and lateral joint laxity using the navigation system, and the amount of tibia cut was decided considering these laxities [11]. The tibial design of Journey TKA system was also asymmetric. Thickness of the thinnest tibial insert was 8.5 mm for the medial side and 11 mm for the latera side. Therefore, for the varus knee, the amount of the bone resection of the lateral tibial plateau was set at 11 mm, and the amount of the resection of the medial side varied from 5 to 9 mm according to the soft tissue balance at this time point (Figs. 1 and 2). For the valgus knee, the amount of the bone resection of the medial tibial plateau was set at 8 mm, and the amount of the resection of the lateral side was decided according to the soft-tissue balance. In the sagittal plane, with use of the navigation system, we reproduce a native slope in patients with a posterior tibial slope of < 10°. In patients with a posterior tibia slope of > 10°, we reduced the posterior slope so as not to exceed 10° [18, 19]. The extension and flexion gaps were measured using a force-controlled, compartment-specific ligament tensioner with a distraction force of 80 N for each of the medial and lateral compartments. For the posterior femur resection, the amount of resection was adjusted to make the extension and flexion gaps of the medial and lateral compartments equal, allowing for a slight lateral ligamentous laxity [20]. For instance, if the joint gap at extension and flexion was 21 mm and 13 mm, respectively, in the medial compartment and 23 mm and 16 mm, respectively in the lateral compartment before the femoral posterior resection, we adjust the cutting amount and the rotation of posterior reference cutting guide to cut 8 mm off the posterior medial femoral condyle and 7 mm off the posterior lateral femoral condyle.

### Medial and lateral joint component gap evaluation

The joint component gap was measured using a femoral trial implant and force-controlled, compartment-specific ligament tensioner [11, 21], with a distraction force of 80 N for both the medial and lateral compartments, at knee flexion of 10°, 30°, 60°, and 90° (Fig. 1). The reason a distraction force of 80N was used in this study was that our previous study which investigated the correlation between the intraoperative component gap at 60 and 80 N and manual mediolateral laxity using the navigation system showed that the component gap at 80 N had a stronger correlation with manual mediolateral laxity than under 60 N. [22]. The patellofemoral joint was reduced during gap measurements. The surgeon performed the measurements twice, and the first assistant surgeon performed them once in 20 randomly selected knees. The intra-rater reliability values at flexion angles of 10°, 30°, 60°, and 90° were 0.97, 0.92, 0.93, and 0.93 for the medial compartment and 0.85, 0.87, 0.82, and 0.86 for the lateral compartment, respectively. The intrer-rater reliability values at flexion angles of 10°, 30°, 60°, and 90° were 0.96, 0.94, 0.91, and 0.88 for the medial compartment and 0.82, 0.79, 0.81, and 0.79 for the lateral compartment, respectively.

### Postoperative rehabilitation

The same rehabilitation protocols were applied in all patients. ROM exercise and walking exercise with a crutch and then a walker were started on the first postoperative day. At 2–3 weeks postoperatively, the patient was discharged from our hospital and completed their rehabilitation protocol with physiotherapists.

### Postoperative evaluation

Regarding radiographic evaluation, coronal plane alignment was measured using full-length standing radiography at postoperative 6-month follow-up. The HKA angle, medial proximal tibial angle (MPTA), and lateral distal femoral angle (LDFA) were measured. The clinical outcomes of PA-BCR TKAs and UKAs were evaluated in terms of the ROM and 2011 KSS at the final follow-up (BCR average, 2.0 years; range, 1–4 years, UKA average, 2.1 years; range, 1–4 years).

### Statistical analysis

Data were analyzed using the Bell Curve 2016 (SSRI Co., Ltd., Tokyo, Japan) software package for Microsoft Windows. A one-way repeated-measure analysis of variance and post hoc pair-wise comparison (Bonferroni test) were used to analyze the join laxity of the medial and lateral compartments at each knee flexion angle. The differences in the joint laxity between the medial and lateral compartments were analyzed using a paired *t*-test. Student’s un-paired t-test was used to compare the quantitative variables and differences between BCR TKAs and UKAs. The estimated sample size was 48 patients to compare the clinical outcomes between BCR TKAs and UKAs according to the statistical power analysis using G*Power 3 (Heinrich Heine Universitat Dusseldorf, FRG) [23]. The effect size used in this study was 0.35. All significance tests were two-tailed, and a significance level of *P* < 0.05 was used for all tests.

## Results

The coronal alignment values of PA-BCR TKAs were as follows: HKA angle, 177.9° ± 2.3° (2.1° in varus); MTPA, 85.4° ± 1.9° (4.6° in varus); and LDFA, 87.5° ± 1.9° (2.5° in valgus).

**Fig. 3:**
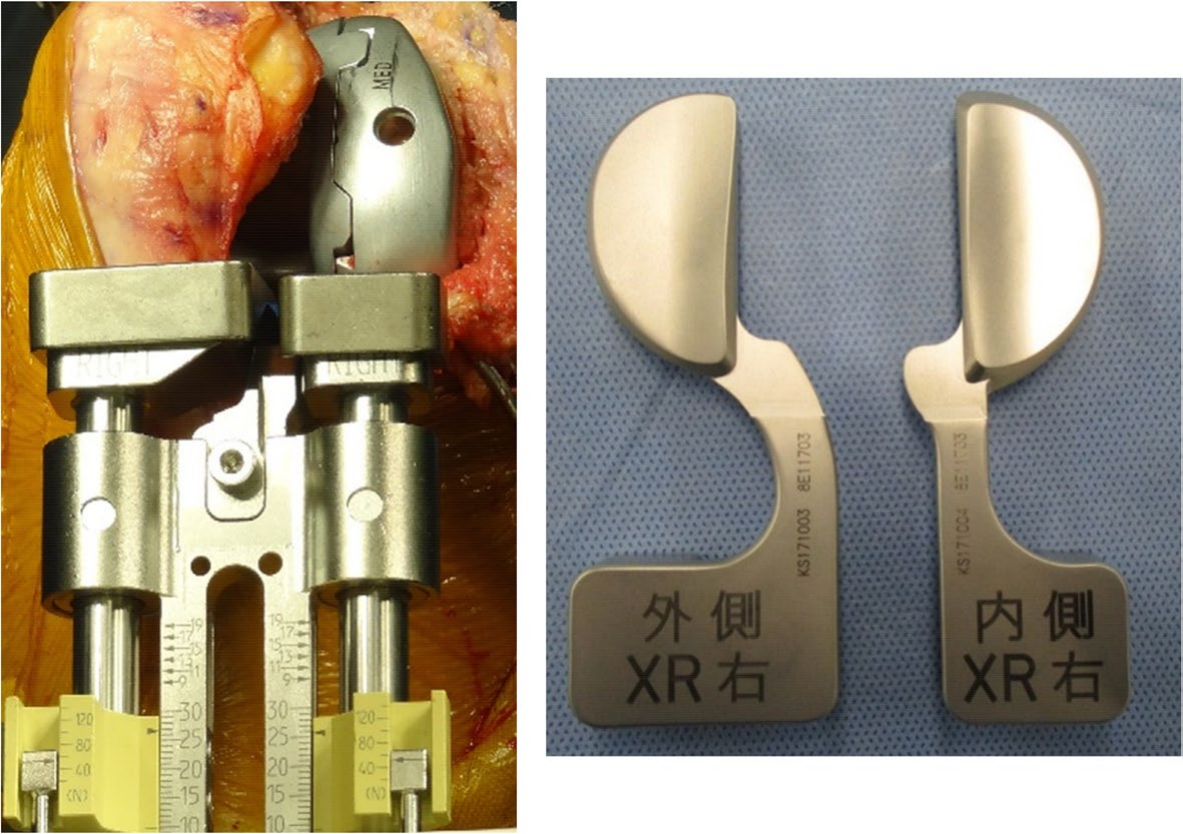
The picture of the tensor device. Measurement of the joint component gap of medial and lateral compartments, respectively, using the tensor device. The upper plates had identical shapes of medial and lateral compartments of the polyethylene surface of the Journey II system

**Fig. 4:**
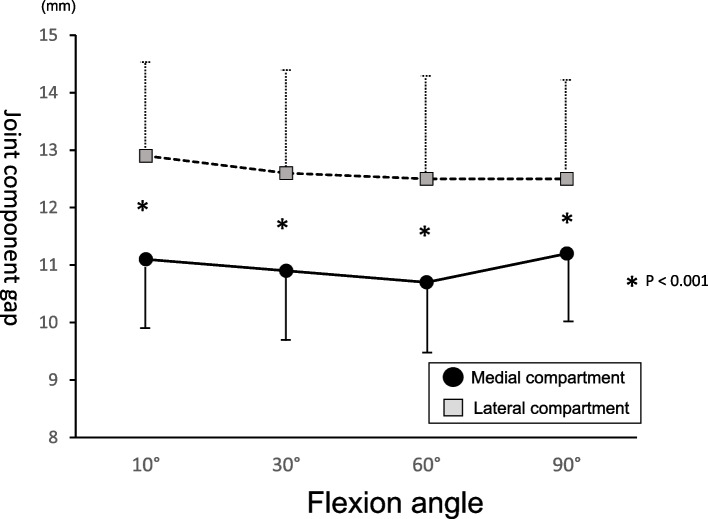
Joint component gap of PA BCR TKA. The joint component gaps of the lateral compartment were significantly larger than those of the medial compartment at each flexion angle. No significant differences were found between the medial and lateral component gaps at each flexion angle. *P < 0.001. FA: functional alignment BCR: bi-cruciate retaining, TKA: total knee arthroplasty

The joint component gaps of the medial and lateral compartments are shown on Figs. 2, 3 and 4. The joint component gaps at flexion angles of 10°, 30°, 60°, and 90° were 11.1 ± 1.2, 10.9 ± 1.4, 10.7 ± 1.3, and 11.2 ± 1.4 mm for the medial compartment and 12.9 ± 1.5, 12.6 ± 1.8, 12.5 ± 1.8, and 12.5 ± 1.7 mm for the lateral compartment, respectively. No significant differences were found between the medial component gaps at each flexion angle. No significant differences were also found between the lateral component gaps at each flexion angle. The joint component gaps of the lateral compartment were significantly larger than those of the medial compartment at each flexion angle (*P* < 0.001). The differences between the medial joint laxity and lateral joint laxity at flexion angles of 10°, 30°, 60°, and 90° were 1.8 ± 1.3, 1.7 ± 1.3, 1.8 ± 1.7, and 1.4 ± 1.7 mm, respectively. No significant differences were found the medial joint component and lateral joint component gaps at each flexion angle.

Table 2 shows the postoperative clinical results. The patient expectation score of PA-BCR TKAs was significantly higher than that of UKAs (*P* = 0.045). The maximum extension angle of PA-BCR TKAs was significantly larger than that of UKAs (*P* = 0.003).

**Table 2:** Postoperative clinical results

	BCR TKA	UKA	*p*-value
Hip-knee-ankle angle (°)	177.9 ± 2.1	177.8 ± 2.7	0.914
Maximum extension (°)	− 0.6 ± 1.5	− 1.8 ± 2.5	0.003
Maximum flexion (°)	127.7 ± 8.7	130.6 ± 9.4	0.153
2011KSS			
Symptom	21.1 ± 3.6	20.8 ± 3.1	0.654
Satisfaction	31.0 ± 8.7	28.4 ± 6.6	0.077
Expectation	10.8 ± 3.0	9.7 ± 2.4	0.045
Activity	78.6 ± 16.7	75.1 ± 15.8	0.278
KOOS			
Pain	89.8 ± 12.4	88.5 ± 13.1	0.589
Symptom	87.1 ± 11.6	88.2 ± 9.2	0.581
ADL	87.8 ± 10.8	86.2 ± 10.7	0.448
Sports	61.9 ± 25.7	60.3 ± 26.2	0.744
QOL	75.3 ± 20.5	68.0 ± 18.5	0.056

*BCR* bi-cruciate retaining, *TKA* total knee arthroplasty, *UKA* unicompartmental knee arthroplasty, *KSS* knee society score, *KOOS* knee injury and osteoarthritis outcome score, *ADL* activities of daily living, *QOL* quality of life

Complications were observed in one case each in the PA-BCR TKA and UKA groups. A complication reported in the BCR TKA group was iliotibial band friction syndrome, which was treated with surgical release of the iliotibial band. A complication in the UKA group was infection, which was treated with debridement and insert change.

## Discussion

The most important finding of the current study is the comparable or a slightly superior short-term clinical outcomes of PA-BCR TKAs to those of UKAs.

The use of PA, a modification of kinematic alignment, might be one of the reasons of the excellent outcomes following BCR TKAs. Soft tissue balancing has been reported to be one of the most important factors for successful TKAs [24–26]. Recently, medial joint stability has been reported to be more important than lateral joint stability for good clinical results and patient satisfaction following ACL-sacrificing TKAs [27, 28]. However, regarding soft tissue balance of BCR TKAs, not only medial joint stability but also lateral joint stability or laxity has been reported to be important [21, 29]. Kaneko et al. showed that postoperative lateral joint stability at 30° and 90° of flexion was associated with better patient expectation [21]. On the contrary, Takasago et al. reported that insufficient lateral joint laxity following BCR TKAs caused kinematic conflict during knee flexion [29]. Therefore, moderate joint laxity would be required for the lateral compartment of BCR TKAs. Kinematic alignment and PA are reported to be superior to mechanical alignment in terms of adjusting the soft tissue balance of both medial and lateral compartments using an ACL-sacrificing TKA prosthesis [12, 30]. In the present study, our PA-BCR TKA technique achieved moderate lateral joint laxity, with joint component gap 1–2 mm larger than the medial compartment at each flexion angle.

Regarding alignment, the HKA angle following PA-BCR TKAs was 177.9°, that is, 2.1° in varus, and was equivalent to the angle following UKAs (177.8°, 2.2° in varus). UKAs were reported to restore the constitutional knee anatomy-like kinematic alignment by the ligament- and bone-sparing methods of UKAs [31]. Therefore, our PA-BCR TKA technique might reproduce constitutional-like limb alignment. With regard to MTPA, Matsumoto et al. investigated the alignment of normal and 454 OA-affected knees of Japanese patients and found that the average tibial plateau inclination was approximately 4° varus in the normal group and those in the early-stage OA group [32]. In the present study, the MTPA after PA-BCR TKA was 85.4° (4.6° in varus). Therefore, the PA-BCR TKA technique might reproduce the constitutional tibial inclination of Japanese patients. About LDFA, most of the reports showed that the average LDFA of Japanese patients were 87 to 88 degrees and almost the same as the current study [33, 34]. There have not been previous reports which showed the normal LDFA of young Japanese people. However Nomoto et al. showed that the LDFA did not differ before and after OA progression even though MPTA decreased significantly from their cohort study [34]. Therefore the LDFA in the current study might also reproduce the constitutional femoral inclination.

These excellent clinical results of PA-BCR TKA in the present study were also thought to be caused by the use of anatomically designed BCR prosthesis. Several studies have demonstrated that knee kinematics after BCR TKAs using non-anatomically designed prosthesis were not the same as normal knees and that ACL forces were higher than that of normal knees [35, 36]. These abnormal kinematics and ligament forces may have contributed to the poor outcomes of BCR TKAs. On the contrary, BCR prosthesis of native knee geometry together with ACL preservation has been reported to provide more normal-like kinematics than contemporary ACL-preserving and ACL-sacrificing prosthesis [37, 38]. The Journey II XR prosthesis system has an anatomical design featuring a tibial baseplate with an asymmetric notch that is positioned more anteriorly on its medial side to accept the ACL footprint and provides greater coverage while not limiting the capacity for rotation. The tibial component also features a non-symmetrical tibial tray with two independently designed medial and lateral inserts. In addition, the femoral component has a kinematic design that can be matched with various tibial baseplate sizes [39].

This study has several limitations. First, this was a retrospective study, not a prospective randomized controlled trial. Second, the follow-up period was relatively short. Further long-term investigations should be performed. Third, preoperative demographics were significantly different between BCR knees and UKA knees. Finally, a relatively small number of patients was evaluated.

## Conclusions

The coronal alignment values of PA-BCR TKA were as follows: HKA angle, 177.9° ± 2.3°; MTPA, 85.4° ± 1.9°; and LDFA, 87.5° ± 1.9°. PA-BCR TKA achieved not only medial stability but also moderate lateral joint laxity. The short-term clinical outcomes of PA-BCR TKA were comparable or a slightly superior to those of UKA.

## Data Availability

The datasets used during the current study are available from the corresponding author on reasonable request.

## Abbreviations

TKA: Total knee arthroplasty
OA: Osteoarthritis
BCR: Bi-cruciate retaining
ACL: Anterior cruciate ligament
PCL: Posterior cruciate ligament
KA: Kinematic alignment
PA: Personalized alignment
UKA: Unicompartmental knee arthroplasty
ON: Osteonecrosis
HKA: Hip knee ankle
ROM: Range of motion
KSS: Knee Society Score
KOOS: Knee injury and osteoarthritis outcome score
MPTA: Medial proximal tibial angle
LDFA: Lateral distal femoral angle
